# TNFα Signaling Regulates Cystic Epithelial Cell Proliferation through Akt/mTOR and ERK/MAPK/Cdk2 Mediated Id2 Signaling

**DOI:** 10.1371/journal.pone.0131043

**Published:** 2015-06-25

**Authors:** Julie X. Zhou, Lucy X. Fan, Xiaoyan Li, James P. Calvet, Xiaogang Li

**Affiliations:** 1 Department of Internal Medicine, University of Kansas Medical Center, Kansas City, KS 66160, United States of America; 2 Kidney Institute, University of Kansas Medical Center, Kansas City, KS 66160, United States of America; 3 Department of Biochemistry and Molecular Biology, University of Kansas Medical Center, Kansas City, KS 66160, United States of America; 4 Department of Anatomy and Cell Biology, University of Kansas Medical Center, Kansas City, KS 66160, United States of America; University of Geneva, SWITZERLAND

## Abstract

Tumor necrosis factor alpha (TNFα) is present in cyst fluid and promotes cyst growth in autosomal dominant polycystic kidney disease (ADPKD). However, the cross-talk between TNFα and PKD associated signaling pathways remains elusive. In this study, we found that stimulation of renal epithelial cells with TNFα or RANKL (receptor activator of NF-κB ligand), a member of the TNFα cytokine family, activated either the PI3K pathway, leading to AKT and mTOR mediated the increase of Id2 protein, or MAPK and Cdk2 to induce Id2 nuclear translocation. The effects of TNFα/RANKL on increasing Id2 protein and its nuclear translocation caused significantly decreased mRNA and protein levels of the Cdk inhibitor p21, allowing increased cell proliferation. TNFα levels increase in cystic kidneys in response to macrophage infiltration and thus might contribute to cyst growth and enlargement during the progression of disease. As such, this study elucidates a novel mechanism for TNFα signaling in regulating cystic renal epithelial cell proliferation in ADPKD.

## Introduction

Autosomal dominant polycystic kidney disease (ADPKD) is caused by mutations in the PKD1 or PKD2 gene [1]. Functional loss of the gene products of PKD1 and PKD2, polycystin 1 and polycystin 2, leads to abnormalities in a variety of intracellular signaling pathways, which contribute to cyst initiation and expansion [2]. In addition to the well-characterized genetic abnormalities, accumulating evidences suggests that inflammation may also play a critical role in cystogenesis [3–6]. Tumor necrosis factor alpha (TNFα), a primary proinflammatory cytokine, is considered to be a potential mediator involved in several kidney diseases, such as renal injury [7] and PKD [3]. The expression of TNFα mRNA is upregulated in *Pkd1* mutant renal epithelial cells and kidney tissues from *Pkd1* knockout mice [4]. TNFα increases progressively with age in cystic kidneys of the rodent ARPKD model, cpk mice, and consistently presents in the cystic fluid from human ADPKD kidneys [8, 9]. TNFα exerts a prosurvival effect on *Pkd1* mutant cystic renal epithelial cells through the activation of NF-κB [4].

Receptor activator of NF-κB ligand (RANKL), a TNF family member, was first found to be a key regulator of osteoblast differentiation and/or activation [10, 11]. RANKL and its receptor RANK have been implicated in the proliferation, survival and differentiation of mammary epithelial cells [12, 13]. RANKL mRNA and protein are detected in the kidney throughout mouse development [14]. A recent study found that the expression of RANKL and RANK in the kidney is increased upon podocyte injury, which acts as the ligand-receptor complex for the survival response during podocyte injury [14]. It has been reported that increased RANKL expression is related to tumor migration and metastasis of renal cell carcinomas [14]. However, the functional role of RANKL in cystic renal epithelial cells has not been determined.

Inhibitor of DNA binding/differentiation 2 (Id2), a member of helix-loop-helix (HLH) family of transcription factors, possesses a HLH motif but lacks the DNA binding domain. Id2 binds to the basic HLH (bHLH) transcription factor to form a heterodimer, which suppresses the functions of bHLH transcription factor in a dominant negative manner [15]. Notably, Id2 acts as a negative regulator of cell differentiation and a positive regulator of cell proliferation mediated by its change in subcellular localization in different cell types. Id2 was seen to be translocated out of the nucleus into the cytosol, leading to the differentiation of oligodendrocytes [16]. However, Id2 was also seen to be translocated into the nucleus, resulting in an increase in cell growth through p21 and the cyclin-dependent kinase (Cdk) Cdk2 in smooth muscle cells [17]. Id2 nuclear localization is triggered by RANKL, which controls cell proliferation of mammary epithelial cells [12].

Increased nuclear localization of Id2 in renal epithelial cells has been reported in kidneys of PKD1 and PKD2 patients, and in *Pkd1* knockout mice [18], which contributes to abnormal epithelial cell proliferation and differentiation in cystic kidneys [18]. Our recent study found that loss of *Pkd1* causes upregulation of Id2 in *Pkd1* mutant mouse embryonic kidney cells, and that knockout of Id2 rescues the renal cystic phenotype of *Pkd1* ^null/null^ mice [19]. However, the mechanism for upregulation of Id2 and its nuclear translocation in *Pkd1* mutant kidneys is unknown and the connection between TNFα and Id2 in renal epithelial cells has not been explored.

We hypothesized that TNFα and RANKL regulated the expression and localization of Id2 in renal epithelial cells, leading to renal epithelial cell proliferation. Our objective is to explore the potential mechanisms involved in regulating this process. In this study, we present that RANKL induces the transcription of TNFα by activating canonical NF-κB signaling in renal epithelial cells. TNFα and RANKL stimulation activates mTOR signaling to increase the expression of Id2, and activate the MAPK-Cdk2 pathway to trigger marked nuclear translocation of Id2, which results in a decrease in p21 expression and an increase in renal epithelial cell growth.

## Materials and Methods

### Cell culture and reagents

*Pkd1* wild type and *Pkd1* null mouse embryonic kidney (MEK) cells, which were generated from Dr. Jing Zhou’s laboratory at Harvard and were used in our recent publications, were maintained as previously described [20]. TNFα, RANKL and rapamycin were purchased from Sigma. Cdk2 inhibitor II, roscovitine, LY294002, SB202190, PD98059, Bay-11-7085, JNK inhibitor and U-46619 were purchased from Calbiochem.

### Extracts of cytoplasmic and nuclear proteins

*Pkd1* wild type MEK cells treated with 20 ng/ml TNFα or 100 ng/ml RANKL for 3 hours were washed by PBS and harvested. The cytoplasmic and nuclear proteins were extracted by the cytoplasmic extract buffer (10 mM HEPES pH 7.9, 10 mM KCl, 0.1 mM EDTA, 0.3% NP-40 with proteinase inhibitor cocktail) and nuclear extract buffer (20 mM HEPES pH 7.9, 0.4 M NaCl, 1 mM EDTA, 25% glycerol with proteinase inhibitor cocktail), respectively. The cytoplasmic and nuclear proteins were analyzed by western blot.

### Western blot

Western blots on whole-cell lysates were performed as previous described [21]. The antibodies used for western blot analysis included: anti-Id2 (Santa Cruz), anti-IκBα (Cell Signaling Technologies) anti-p21 (Santa Cruz), anti-AKT (Cell Signaling Technologies), anti-phospho-Akt (Cell Signaling Technologies), anti-S6 (Cell Signaling Technologies), anti-phospho-S6 (Cell Signaling Technologies), anti-mTOR (Cell Signaling Technologies), anti-ERK (Cell Signaling Technologies), anti-phospho-ERK (Cell Signaling Technologies), anti-Cdk2 (Santa Cruz), anti-phospho-Cdk2 (Cell Signaling Technologies), anti-Lamin A/C (Cell Signaling Technologies) and anti-actin (Sigma). Donkey-anti-rabbit IgG-horseradish peroxidase and Donkey-anti-mouse IgG-horseradish peroxidase (Santa Cruz) were used as secondary antibodies. The band intensities were analyzed by NIH Image J.

### MTT assay

The effects of TNFα and RANKL on cell viability were determined using the 3-(4,5-dimethylthiazol-2-yl)-2,5-diphenyltetrazolium bromide (MTT) assay. *Pkd1* wild type MEK cells (5 × 104/mL, 200 μL) were cultured in a 96-well plate overnight and stimulated with TNFα (20 ng/ml) or RANKL (100 ng/ml) for 24 hours, respectively. Twenty microliters MTT was added to each well, and incubated for 4 hours. After removal of the medium, 150 μL dimethyl sulfoxide (DMSO) was added to each well and the absorbance at 490 nm was analyzed. The cell viability of untreated cells was normalized to one.

### Quantitative reverse-transcription polymerase chain reaction

Total RNA extracted by the RNeasy plus mini kit (Qiagen) was used to synthesize cDNA as previous described [22]. RNA expression profiles were analyzed by real-time PCR using iTaq SYBER Green Supermix (BioRad) in a CFX Connect System. Genes were amplified using the following primers. TNFα-F: 5′-CTTCTGTCTACTGAACTTCGGG-3′; TNFα-R, 5′-CAGGCTTGTCACTCGAATTTTG-3′; Id2-F: 5′-TGAACACGGACATCAGCATC-3′; Id2-R: 5′-AAGAAAAAGTCCCCAAATGCC-3′; p21-F: 5′- CAGATCCACAGCGATATCCAG -3′; p21-R: 5′-AGAGACAACGGCACACTTTG -3′; Actin-F: 5′-AAGAGCTATGAGCTGCCTGA-3′; Actin-R: 5′-TACGGATGTCAACGTCACAC-3′. The complete reactions were subjected to the following program of thermal cycling: 40 cycles of 10s at 95°C and 20s at 61°C, a melting curve was run after the PCR cycles, followed by a cooling step. Each sample was run in triplicate in each experiment. The expression levels of TNFα, Id2 and p21 were normalized to the expression level of actin.

### RNA interference

The RNA oligonucleotides that specifically target mouse Id2 were purchased from Thermo Dharmacon. The RNA oligonucleotides were transfected with the DharmaFECT siRNA transfection reagent (Dharmacon).

### Immunofluorescence

*Pkd1* wild type MEK cells treated with TNFα and RANKL were grown on glass coverslips, rinsed with 1× phosphate-buffered saline (PBS), fixed with 4% paraformaldehyde containing 2% sucrose for 10 min, and permeabilized with 1% Triton X-100 in PBS for 5 min. Anti-Id2 (Santa Cruz, 1:100 dilution), was used for cell staining. Fluro 488-conjugated anti-rabbit IgG antibody (Invitrogen) was used at a dilution of 1:10000. Prolong anti-fade reagent (Invitrogen) was used with DAPI. Immunofluorescence images were obtained with a NIKON ECLIPSE 80i Microscope.

### Data analysis

All quantifiable data are reported as mean ± SEM. Comparisons between two groups were carried out using an unpaired 2-tailed Student’s t test. Differences in the variables between multiple groups were determined by one-way ANOVA post hoc test. The differences between two groups were statistically significant at P values less than 0.05.

## Results

### RANKL induces TNFα transcription in renal epithelial cells

**Fig 1:**
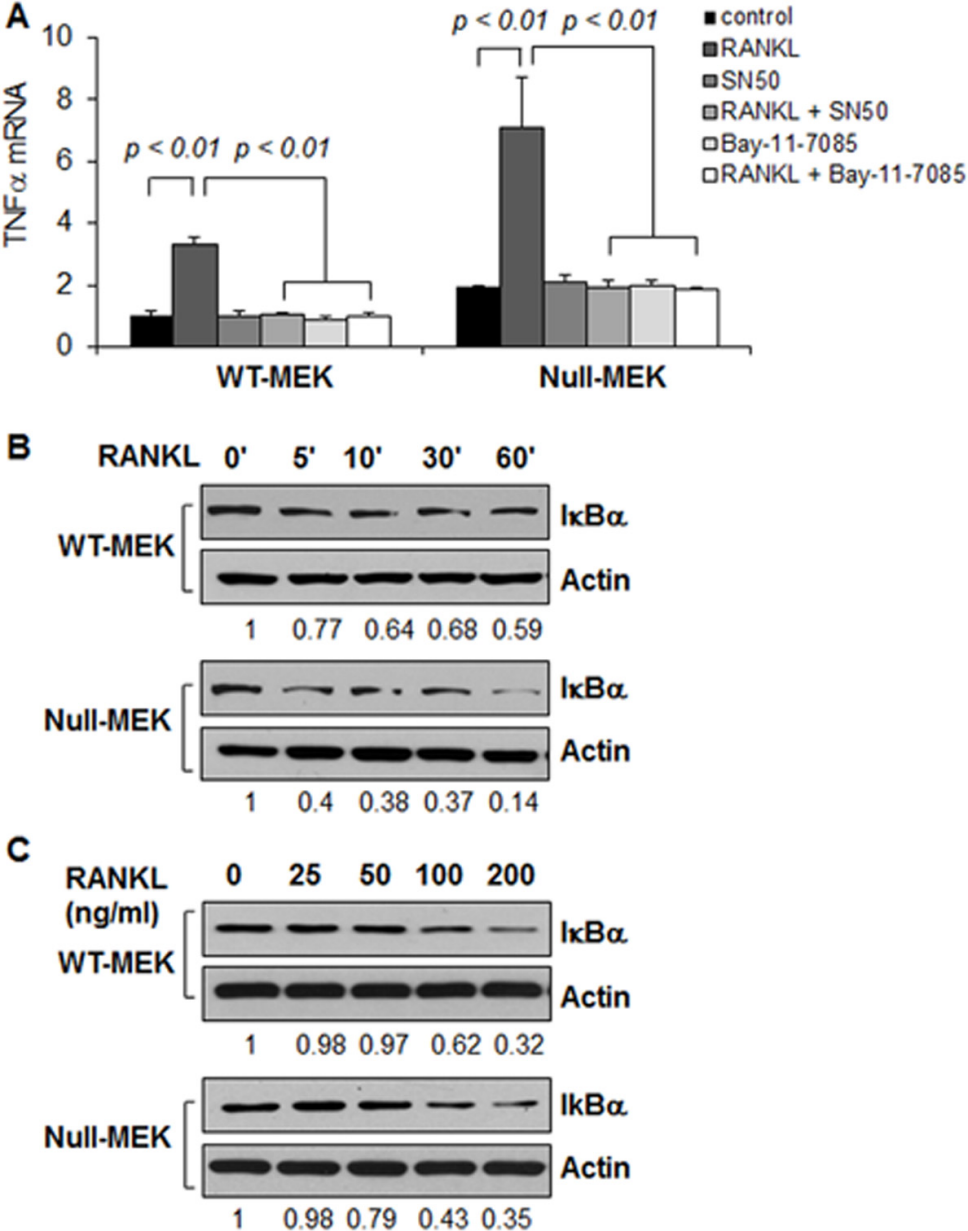
RANKL stimulation increases the expression of TNFα in renal epithelial cells. (A) The mRNA levels of TNFα in *Pkd1* wild type and *Pkd1* null MEK cells treated with RANKL, SN50, RANKL plus SN50, Bay-11-7085, Bay-11-7085 plus RANKL and vehicle control, respectively, for 6 hours were analyzed by qRT-PCR. The expression levels of TNFα were normalized to the expression levels of actin. n = 3, ANOVA, p < 0.01. (B and C) Western blot analysis of the expression of I-κBα from whole cell lysates of *Pkd1* wild-type and *Pkd1* null MEK cells treated with RANKL (100 ng/ml) at indicated time point (B) and at indicated concentration for 45 mins (C). The numbers at the bottom indicate the relative intensities of the bands, which are normalized to Actin.

Our previous studies showed that TNFα mRNA was increased in *Pkd1* mutant renal epithelial cells, and that TNFα mRNA induces its own transcription through activating canonical NF-κB signaling [4]. RANKL, a TNFα family molecule, can also activate NF-κB signaling and transcriptionally regulate TNFα in osteoclasts and cancer cells [23, 24]. Therefore, we examined whether RANKL also regulated TNFα transcription in renal epithelial cells. We found that TNFα mRNA was increased in response to RANKL stimulation in *Pkd1* wild type and mutant mouse embryonic kidney (MEK) cells as analyzed by quantitative reverse transcription PCR (qRT-PCR) (Fig 1A). RANKL treatment decreased the expression of IκBα in a time-dependent (Fig 1B) and dose-dependent manner (Fig 1C), suggesting the activation of NF-κB signaling. We further found that two NF-κB inhibitors, SN50 and Bay-11-7085 [25], blocked the upregulation of TNFα mRNA induced by RANKL (Fig 1A), suggesting that RANKL induced TNFα upregulation is mediated by NF-κB signaling.

### TNFα and RANKL increased protein expression of Id2 in renal epithelial cells

**Fig 2:**
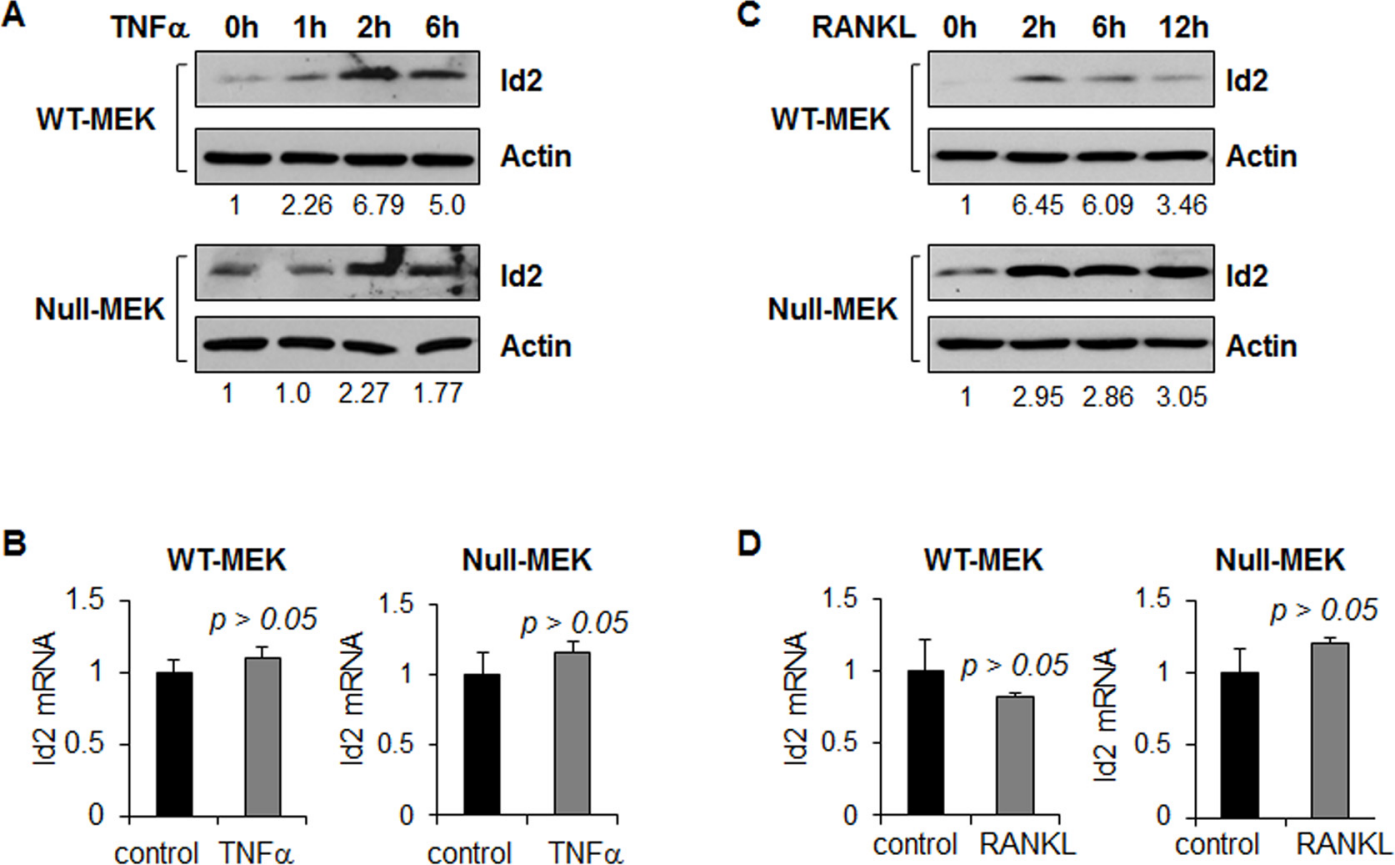
TNFα and RANKL stimulation increase the expression of Id2 protein in renal epithelial cells. (A and B) the expression levels of Id2 protein (A) and mRNA (B) in *Pkd1* wild type and *Pkd1* null MEK cells treated with TNFα (20 ng/ml) analyzed by western blot and qRT-PCR, respectively. (C and D) the expression levels of Id2 protein (C) and mRNA (D) in *Pkd1* wild type and *Pkd1* null MEK cells treated with RANKL (100 ng/ml) analyzed by western blot and qRT-PCR, respectively. The numbers at the bottom indicate the relative intensities of the bands, which are normalized to actin.

Id2 has been found to be upregulated in *Pkd1* mutant renal epithelial cells [19]. To investigate whether TNFα and RANKL regulate the expression of Id2 in renal epithelial cells, we treated *Pkd1* wild type and *Pkd1* mutant MEK cells with TNFα and RANKL, respectively. We found that TNFα and RANKL treatment increased the expression of Id2 in these cells as analyzed by western blot (Fig 2A and 2C). However, TNFα and RANKL treatment did not affect the Id2 mRNA expression as analyzed by qRT-PCR (Fig 2B and 2D). These results suggest that TNFα and RANKL regulate the expression of Id2 at the translational level but not transcriptionally.

### TNFα and RANKL regulate the expression of Id2 through activation of the Akt/mTOR pathway

**Fig 3:**
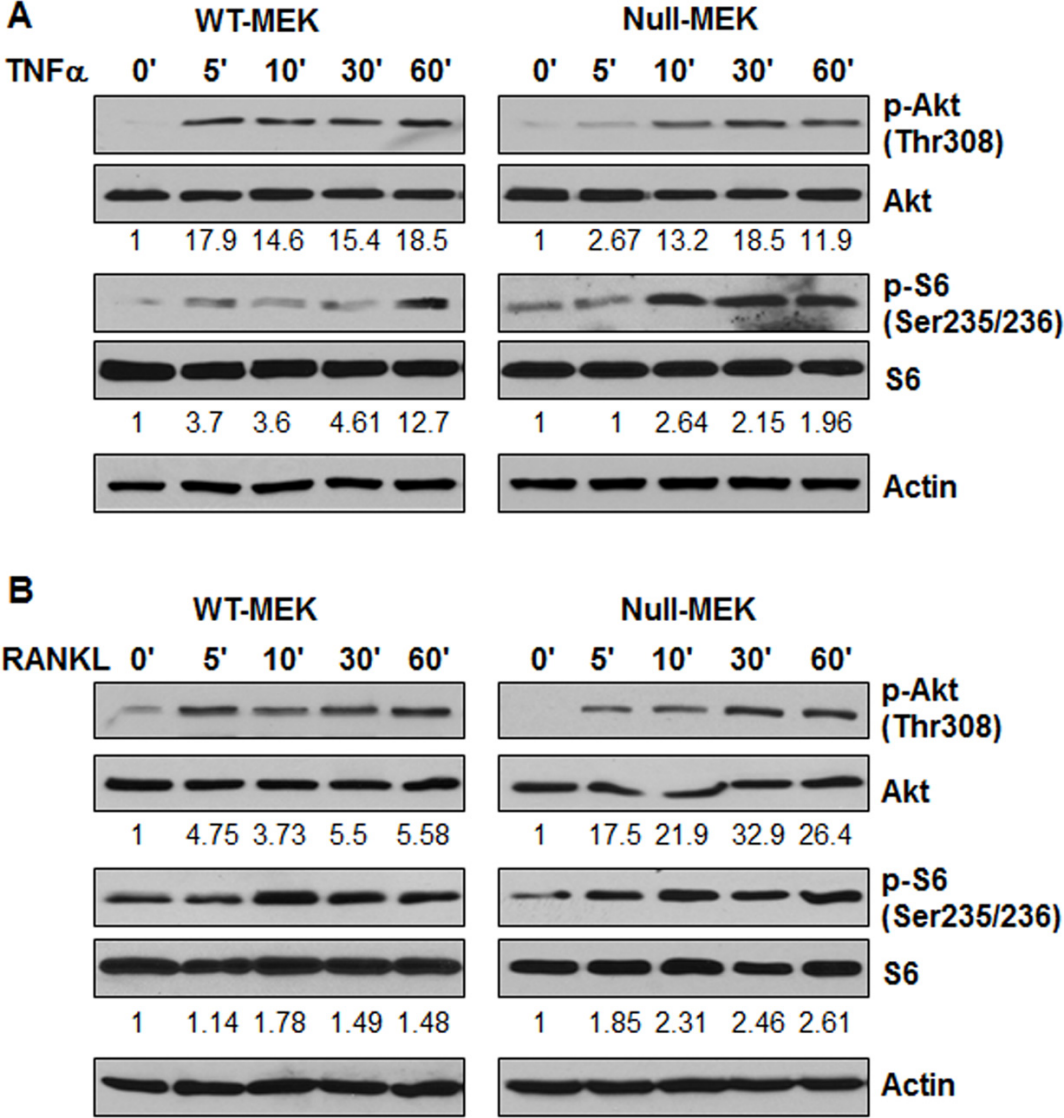
TNFα and RANKL stimulation activate the Akt and mTOR pathway. Western blot analysis of the expression of phospho-Akt, Akt, phospho-S6, and S6 from whole cell lysates of *Pkd1* wild-type and *Pkd1* null MEK cells treated with TNFα (20 ng/ml) (A) and RANKL (100 ng/ml) (B). The numbers at the bottom indicate the intensities of p-Akt relative to total Akt and p-S6 relative to total S6.

TNFα and RANKL have been found to activate PI3K in osteoclasts [26]. Activation of PI3K allows phosphoinositide-dependent kinase 1 (PDK1) to access and phosphorylate Thr308 in the activation loop of Akt [27]. The activation of Akt results in the phosphorylation and activation of mTOR in cancer and PKD [28, 29]. The activated mTOR has been found to regulate Id2 expression in mammary epithelial cells [30]. We found that TNFα and RANKL treatment induced the phosphorylation of Thr308 of Akt in *Pkd1* wild type and mutant MEK cells (Fig 3A and 3B). Phosphorylation of Akt activates mTOR, which induces the activation of p70 S6 kinase at and the subsequent phosphorylation of S6 ribosomal protein [31]. We found that TNFα and RANKL treatment also induced phosphorylation of S6 in *Pkd1* wild type and mutant MEK cells (Fig 3A and 3B).

**Fig 4:**
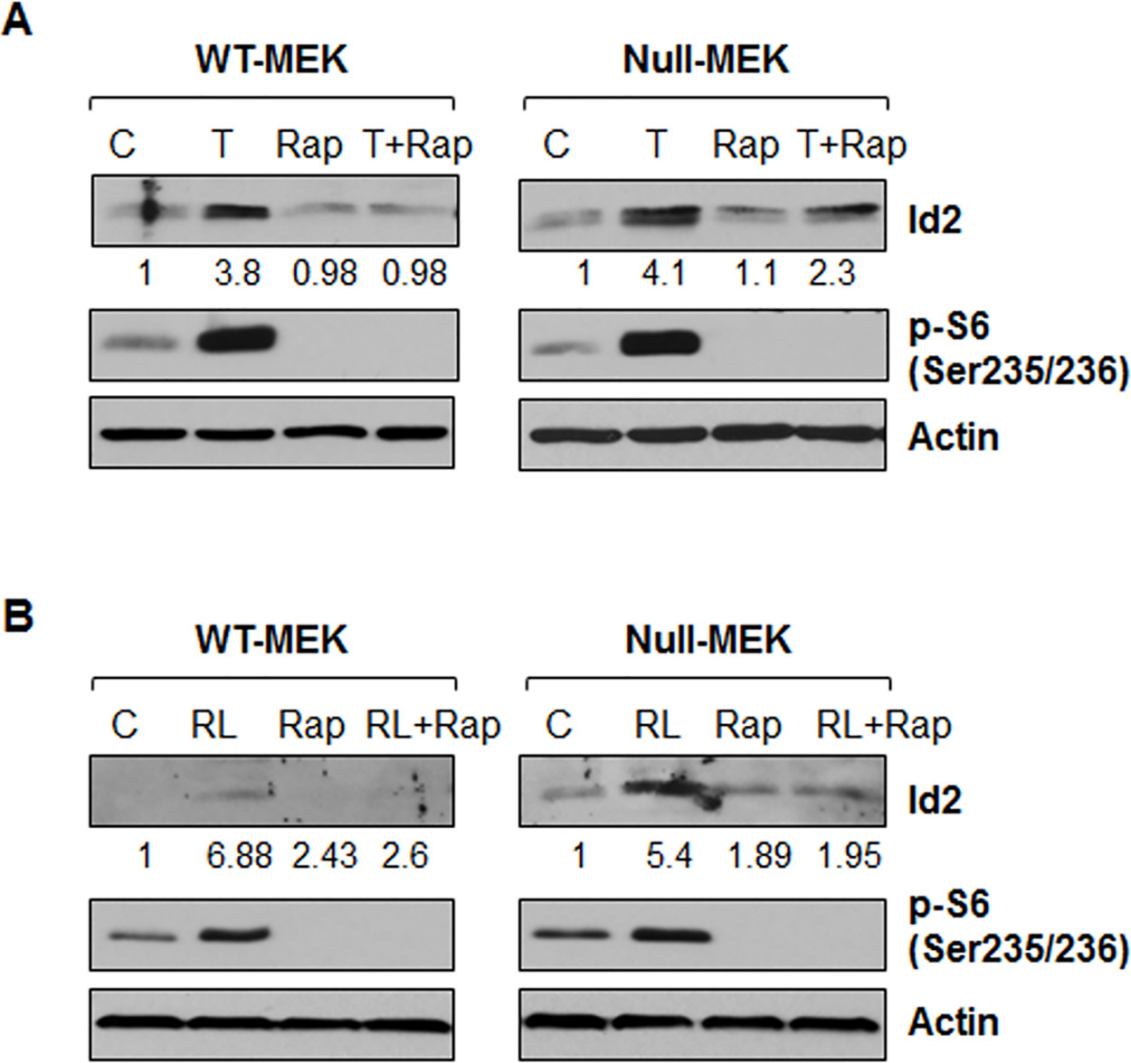
Inhibition of mTOR with rapamycin blocks the upregulation of Id2 induced by TNFα and RANKL. *Pkd1* wild-type and *Pkd1* null MEK cells were not treated (C) or treated with TNFα (T) (20 ng/ml) (A), RANKL (100 ng/ml) (B) in the absence or presence (Rap) of rapamycin (10 nM) for 3 hours. The expression of Id2 and phospho-S6 (p-S6) were analyzed by western blot. The numbers at the bottom indicate the relative intensities of the Id2 bands, which are normalized to actin.

Next, we examined the relationship between mTOR activation and Id2 expression upon TNFα and RANKL treatment in *Pkd1* wild type and mutant MEK cells. For this purpose, *Pkd1* wild type and mutant MEK cells were induced with TNFα or RANKL in the absence and presence of rapamycin, and Id2 protein levels were analyzed by western blot. We found that rapamycin completely blocked the induction of Id2 protein in these cells upon TNFα and RANKL treatment (Fig 4). These results suggested that activation of mTOR contributes to Id2 upregulation induced by TNFα and RANKL in renal epithelial cells.

**Fig 5:**
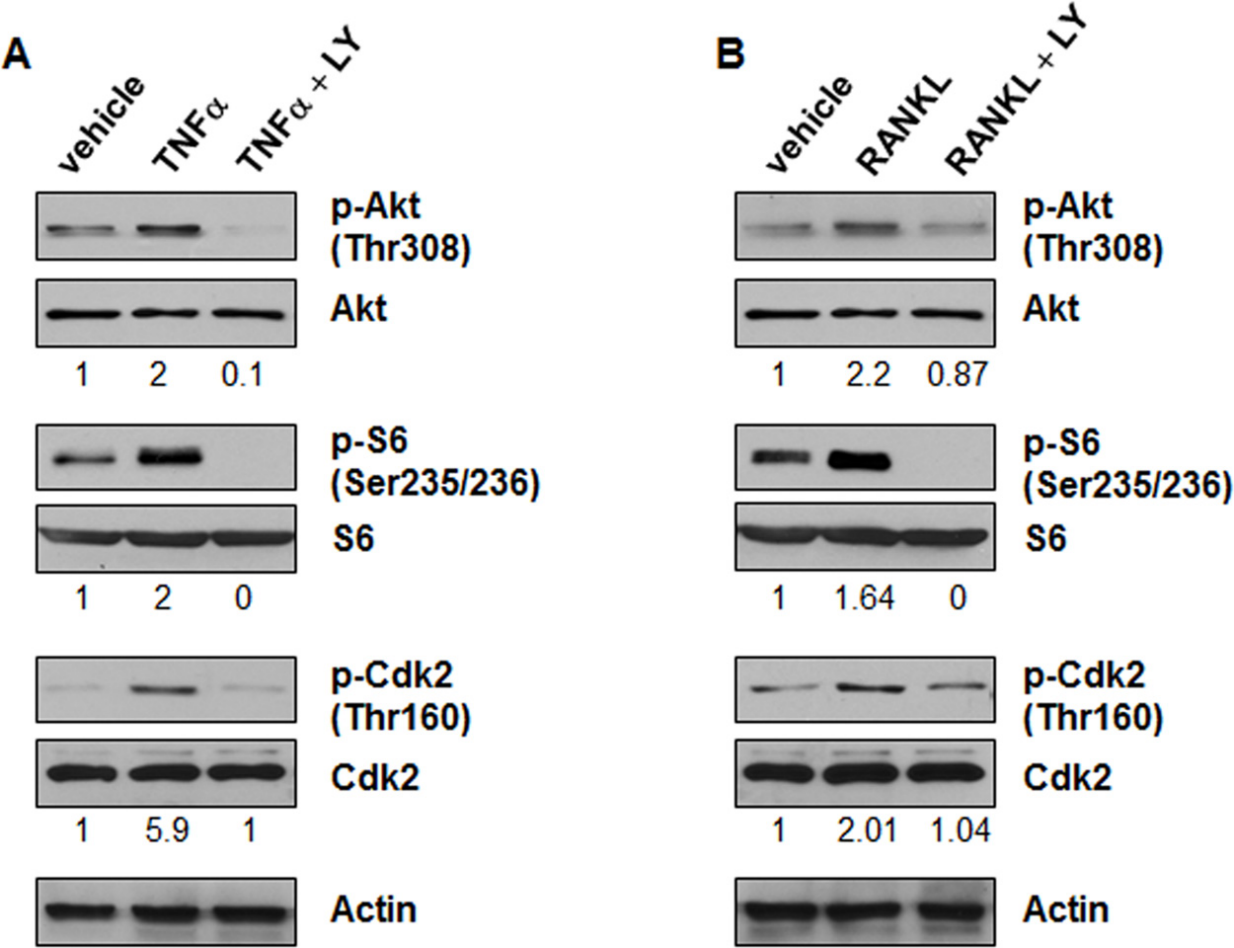
Inhibition of PI3K blocks the activation of mTOR and Cdk2 induced by TNFα and RANKL. *Pkd1* wild type MEK cells were pretreated with PI3K inhibitor, LY294002 (20 μM, mark as LY), for 1 hour, and then stimulated by TNFα (20 ng/ml) (A) or RANKL (100 ng/ml) (B) for 30 minutes. The expression of phospho-Akt, Akt, phospho-S6 and S6, phospho-Cdk2 and Cdk2 from whole cell lysates was analyzed by western blot. The numbers at the bottom indicate the intensities of p-Akt relative to total Akt, p-S6 relative to total S6 and p-Cdk2 relative to total Cdk2.

Last, we found that treatment with TNFα or RANKL in the presence of LY294002, an inhibitor of PI3K, decreased the phosphorylation of AKT and S6 but not the levels of AKT and S6 in *Pkd1* wild type MEK cells compared with cells treated with TNFα or RANKL alone (Fig 5A and 5B). These results suggested that TNFα and RANKL regulate the expression of Id2 through PI3K to activate the Akt/mTOR pathway.

### TNFα and RANKL induce Id2 nuclear translocation in renal epithelial cells

**Fig 6:**
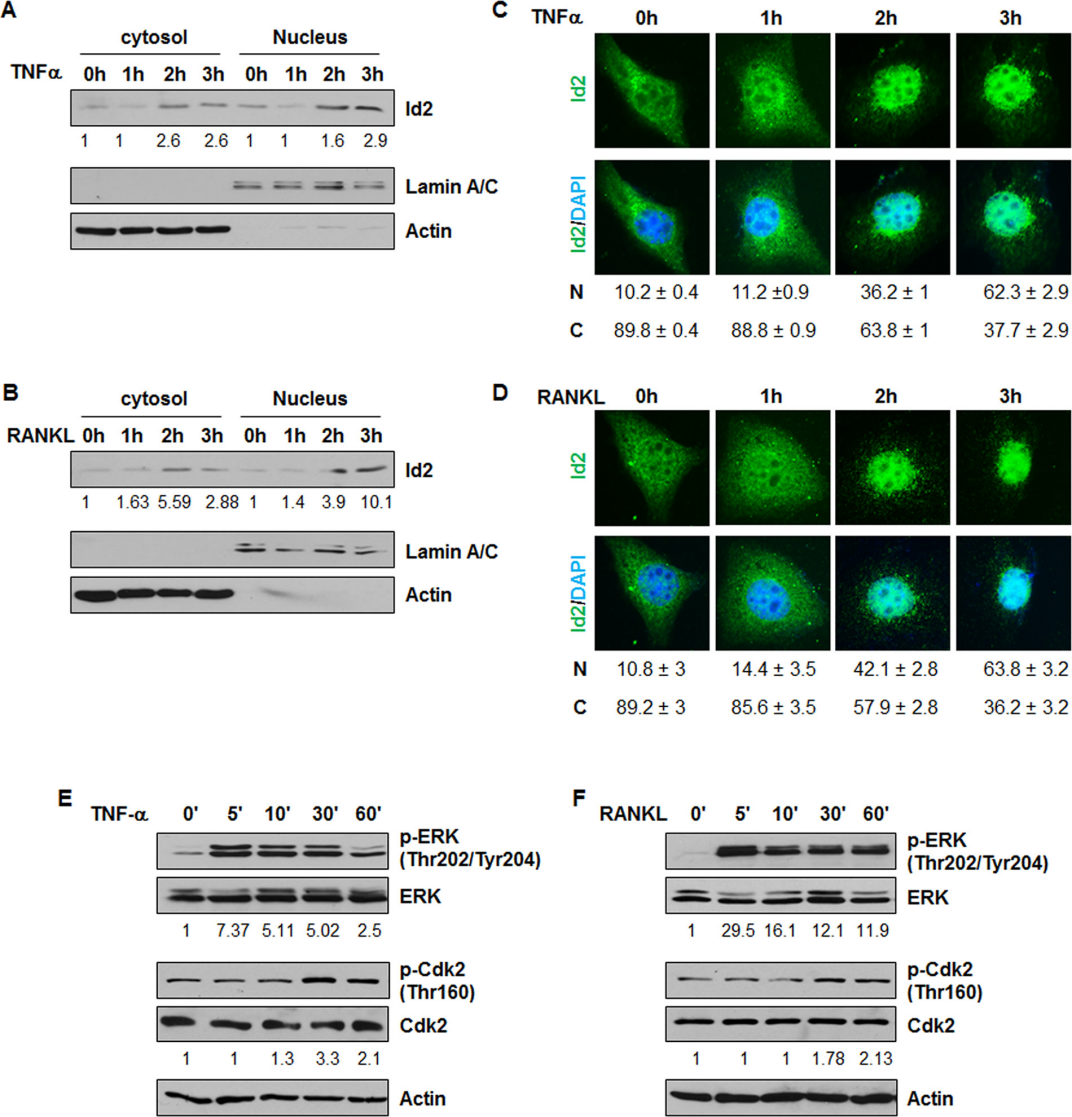
TNFα and RANKL stimulation increases the nuclear translocation of Id2 in *Pkd1* wild type MEK cells. (A-B) Western blot analysis of the expression of Id2 from cytoplasmic and nuclear fractions of *Pkd1* wild-type MEK cells treated with TNFα (A) and RANKL (B). The numbers at the bottom indicate the intensities of Id2 bands relative to actin and Lamin A/C in cytoplasmic fractions and nuclear fractions, respectively. (C-D) Localization of Id2 in *Pkd1* wild type MEK cells after TNFα (C) and RANKL (D) treatment at the indicated time point. Id2 was detected with an anti-Id2 antibody, followed by Fluro 488 conjugated anti-rabbit IgG antibody (Green). Nuclear DNA was stained with DAPI (Blue). Images represented one of three independent experiments. Percentage of cells with nuclear (N) and cystoplasmic (C) localization are indicated below the images. (E-F) Western blot analysis of the expression of phospho-ERK, ERK, phospho-Cdk2 and Cdk2 from whole cell lysates of *Pkd1* wild-type MEK cells treated with TNFα (20 ng/ml) (E) and RANKL (100 ng/ml) (F). The numbers at the bottom indicate the intensities of p-ERK relative to total ERK, and p-Cdk2 relative to total Cdk2.

Enhanced Id2 nuclear localization was found in human and mouse cystic kidneys [18]. It has been reported that RANKL triggers marked nuclear translocation of Id2 in mammary epithelial cells [12]. However, whether or not TNFα is able to stimulate Id2 nuclear translocation is unknown. As RANKL belongs to the TNF family and TNFα has been found to exist in cyst fluid of ADPKD mice and patients [3], we have proposed that TNFα and RANKL regulate Id2 nuclear translocation in renal epithelial cells. In support of this, we found that TNFα or RANKL treatment increased Id2 expression not only in whole cell lysates (Fig 2A and 2C) but also in both the cytosolic and nuclear fractions in *Pkd1* wild type MEK cells (Fig 6A–6D) as examined by western blot and immunostaining.

**Fig 7:**
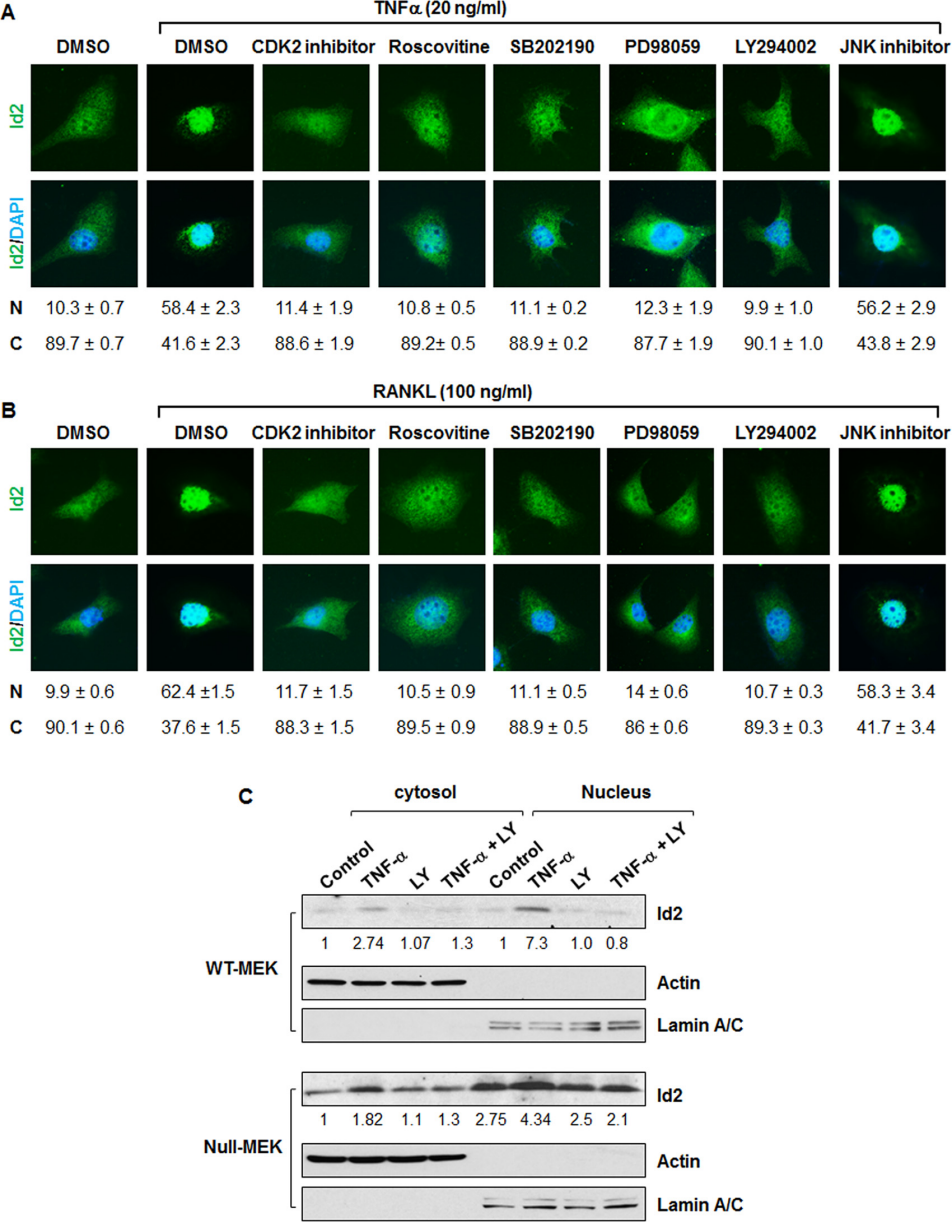
Inhibition of Cdk2 blocks nuclear translocation of Id2 induced by TNFα and RANKL. Localization of Id2 in *Pkd1* wild type MEK cells after TNFα (A) and RANKL (B) treatment in the presence of kinase inhibitors. *Pkd1* wild type MEK cells were placed in DMEM F12 containing 1% FBS for 12 hours. Serum starved cells were treated with DMSO or the indicated kinase inhibitors for 3 hours. Inhibitor pre-treated cells were untreated or stimulated with TNFα and RANKL for 3 hours. Id2 was detected with an anti-Id2 antibody, followed by Fluro 488 conjugated anti-rabbit IgG antibody (Green). Nuclear DNA was stained with DAPI (Blue). One result, representative of three independent experiments, is shown. Percentage of cells with nuclear (N) and cystoplasmic (C) localization are indicated below the images. (C) Western blot analysis of the expression of Id2 from cytoplasmic and nuclear fractions of *Pkd1* wild-type and null MEK cells treated with TNFα, LY294002 (LY), TNFα plus LY294002, and vehicle control for 3 h. The numbers at the bottom indicate the intensities of Id2 bands relative to actin and Lamin A/C in cytoplasmic fractions and nuclear fractions, respectively.

Previous studies reported that cyclin E/Cdk2 phosphorylates serine 5 of Id2 [32] and phosphorylation of Id2 is required for its nuclear retention in mammary epithelial cells [13]. We found that TNFα or RANKL treatment induced the phosphorylation of Cdk2 (Fig 6E and 6F). Consistent with the report that phosphorylation Cdk2 is regulated by phosphorylation and activation of ERK in mammary epithelial cells [12], we found that treatment with TNFα or RANKL also induced phosphorylation of ERK in renal epithelial cells (Fig 6E and 6F). We further found that treatment with TNFα or RANKL plus Cdk2 inhibitor II or roscovitine significantly blocked Id2 nuclear translocation in renal epithelial cells (Fig 7A and 7B), suggesting that Cdk2 is required for the TNFα or RANKL induced nuclear translocation of Id2 in these cells. It has been reported that PI3K regulates the phosphorylation of Cdk2 [33]. We found that co-treatment with a PI3K inhibitor (LY294002) plus TNFα or RANKL decreased the phosphorylation of Cdk2 in *Pkd1* wild type and mutant MEK cells compared to cells treated with TNFα or RANKL alone (Fig 5A and 5B). MAPK signaling has also been reported to regulate the activation of Cdk2 [34]. To further investigate the involvement of PI3K, MAPK, and JNK in TNFα or RANKL induced Id2 nuclear translocation, *Pkd1* wild type MEK cells were treated with different inhibitors prior to TNFα or RANKL stimulation, including the PI3K inhibitor LY294002, p38 inhibitor SB202190, ERK inhibitor PD98059 and JNK inhibitor. We found that inhibition of PI3K and MAPK, but not inhibition of JNK, abrogated the nuclear translocation of Id2 induced by TNFα or RANKL stimulation (Fig 7A and 7B). These results suggested that TNFα or RANKL induced nuclear translocation of Id2 might be through PI3K and MAPK mediated phosphorylated Cdk2. In addition, we found that treatment with PI3K inhibitor LY294002 blocked TNFα induced the upregulation of Id2 in nuclear and cytosol fraction in both *Pkd1* wild type and null MEK cells (Fig 7C).

### TNFα and RANKL repress p21 expression mediated by Id2 in renal epithelial cells

**Fig 8:**
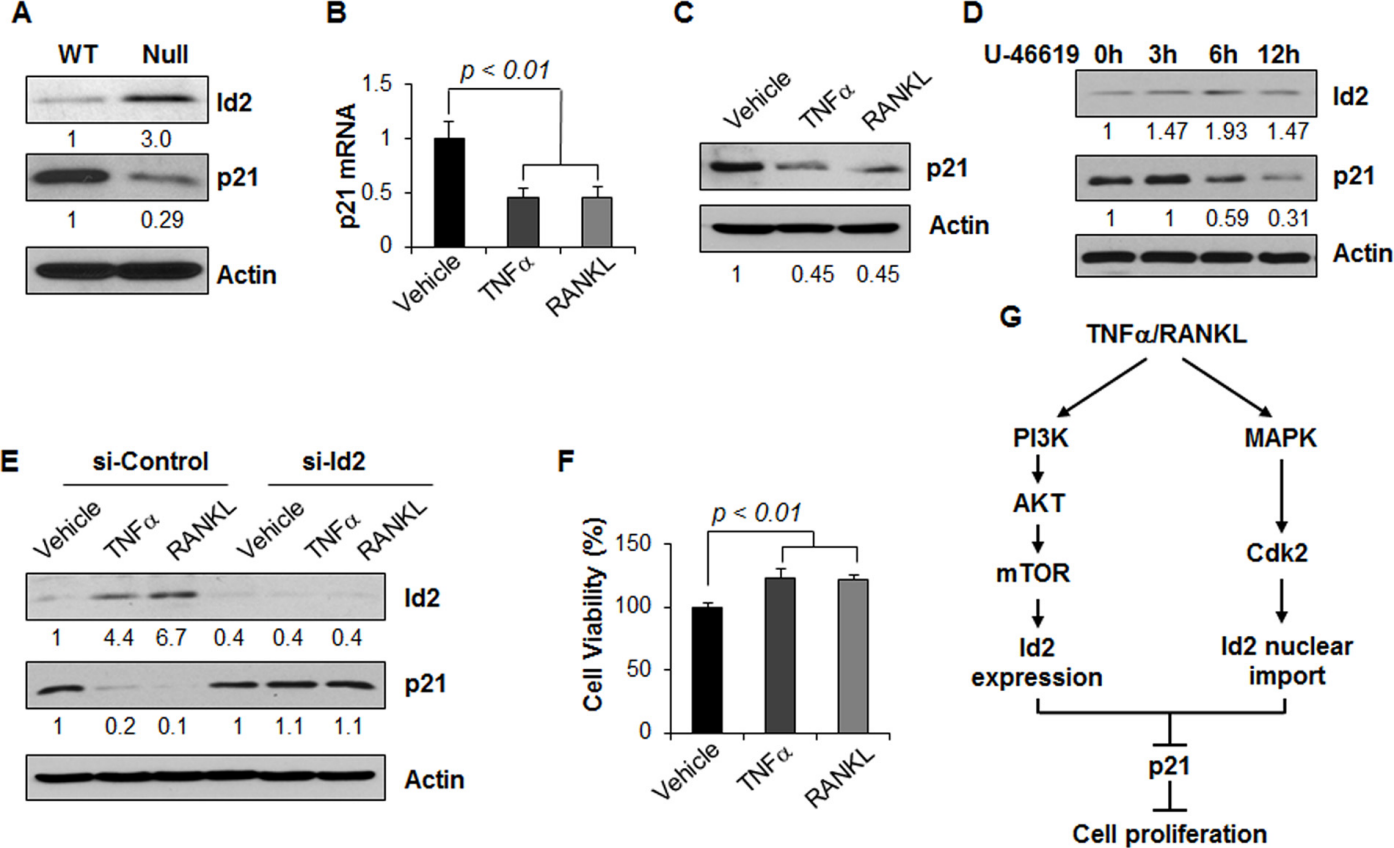
TNFα and RANKL stimulation regulates the expression of p21 in *Pkd1* wild type MEK cells. (A) Western blot analysis of the expression of Id2 and p21 from whole cell lysates of *Pkd1* wild type and null MEK cells. The numbers at the bottom indicate the relative intensities of the bands, which are normalized to actin. (B and C) TNFα and RANKL stimulation decreased the expression of p21 mRNA (B) and protein (C). n = 3, ANOVA, p < 0.01. The numbers at the bottom indicate the relative intensities of the bands, which are normalized to actin. (D) Western blot analysis of the expression of Id2 and p21 from whole cell lysates of *Pkd1* wild type MEK cells treated with p38 activator U-46619 (5 μM). The numbers at the bottom indicate the relative intensities of the bands, which are normalized to actin. (E) *Pkd1* wild type MEK cells were transfected with control siRNA or Id2 siRNA for 24 hours, and then treated with TNFα and RANKL for 24 hours. The expression of Id2 and p21 in these cells were analyzed by western blot. The numbers at the bottom indicate the relative intensities of the bands, which are normalized to actin. (F) TNFα and RANKL stimulation increased the proliferation of *Pkd1* wild type MEK cells analyzed by MTT assay. n = 4, ANOVA, p < 0.01. (G) TNFα and RANKL mediated pathways in renal epithelial cells.

Our previous studies found that Id2 regulates the cell cycle through downregulation of p21, an inhibitor of CDK2, in cystic renal epithelial cells [18]. Id2 was upregulated, and p21 was downregulated in *Pkd1* null MEK cells compared with *Pkd1* wild type MEK cells (Fig 8A). As TNFα or RANKL increases the expression and nuclear translocation of Id2, next, we examined the expression of p21 in *Pkd1* wild type MEK cells upon TNFα or RANKL treatment by qRT-PCR and western blot. We found that treatment with TNFα or RANKL decreased the expression of p21 mRNA (Fig 8B) and protein levels (Fig 8C) in *Pkd1* wild type MEK cells. We further found that treatment with an activator of p38, U-46619, increased the expression of Id2 but decreased the expression of p21 in *Pkd1* wild type MEK cells (Fig 8D).

To further confirm that the downregulation of p21 induced by TNFα or RANKL stimulation is mediated by Id2, *Pkd1* wild type MEK cells were transfected with Id2 siRNA and then were treated by TNFα or RANKL, respectively. We found that TNFα or RANKL decreased p21 expression in the control siRNA transfected cells, but not in the Id2 siRNA transfected cells (Fig 8E). We further found that TNFα or RANKL treatment increased renal epithelial cell growth as analyzed by MTT assay (Fig 8F). These results suggested that TNFα or RANKL can regulate renal epithelial cell proliferation via Id2-p21 signaling (Fig 8G).

## Discussion

TNFα is considered to be a critical effector cytokine in ADPKD, and anti-TNFα therapy and targeting TNFα dependent signaling pathways have emerged in preclinical studies to have high potential in the treatment of this disease [3, 4]. In this study, we integrate TNFα signaling with other PKD associated pathways in regulating cystic renal epithelial cell proliferation. We found that: 1) TNFα signaling regulates the phosphorylation and activation of mTOR, leading to upregulation of Id2 in *Pkd1* wild type and mutant MEK cells; 2) TNFα signaling regulates the phosphorylation and activation of ERK/MAPK and Cdk2, leading to Id2 nuclear translocation in *Pkd1* wild type renal epithelial cells; 3) TNFα signaling mediates upregulation of Id2 and increased nuclear import of Id2, p21 inhibition, and increases in renal epithelial cell proliferation; and 4) RANKL, a TNF family molecule, is able to regulate the transcription of TNFα in renal epithelial cells via activation of NF-αB. The effects of TNFα and RANKL mediated through PI3K, Akt/mTOR, and MAPK/Cdk2 to Id2 translation, Id2 nuclear import, and p21 are summarized in Fig 8G.

In ADPKD kidneys and *Pkd1* mutant mouse kidneys, Id2 was found to be upregulated and in both the cytosol and nucleus instead of in its normal cytoplasmic localization [18]. Id2 nuclear import inhibits p21 and increases proliferation of cystic epithelial cells [18]. Targeting Id2 with RNAi normalized the cell cycle profile of *Pkd1* mutant MEK cells [18]. Consistent with this, Id2-Pkd1 double knockout rescues the renal cystic phenotype [19]. The mechanism(s) underlying regulation of the elevated Id2 expression and nuclear transport in cystic epithelial cells has been discussed [18, 19]. In this study, we present for the first time that TNFα regulates the expression and nuclear translocation of Id2 in renal epithelial cells, suggesting that cyst fluid TNFα may contribute to the upregulation and increased nuclear ld2 in ADPKD kidneys and *Pkd1* mutant mouse kidneys.

It has been reported that TNFα signaling through IKK complexes activate NF-κB, leading to subsequent activation NF-κB-dependent gene transcription [35]. To further investigate whether the elevated expression of Id2 by TNFα is through the NF-κB-dependent pathway, we used specific inhibitors of NF-κB, including the NF-κB nuclear translocation inhibitor SN50 (Calbiochem) [36], and then examined the expression of Id2 in TNFα treated or untreated *Pkd1* null MEK cells. However, our results suggested that TNFα induced Id2 upregulation was not through the activation of NF-κB (data not shown). Other mechanisms are involved in this process.

It has been found that TNFα can activate the mTOR pathway [37], and that mTOR regulates the functional differentiation of mammary epithelial cells through Id2 [30]. The mTOR pathway is inappropriately activated in cyst-lining epithelial cells in human ADPKD patients and mouse models [38]. Inhibition of mTOR with rapamycin has been shown to reverse cystogenesis in PKD kidneys [38]. However, whether TNFα signaling is able to activate mTOR and how the activated mTOR might regulate cystic epithelial cell proliferation and differentiation has remained unknown. Based on the fact that mTOR activation is represented by the phosphorylation status of S6K1 at T389, a well-known mTOR downstream effector [39], we found that TNFα treatment was indeed able to induce this phosphorylation in *Pkd1* wild-type and null MEK cells (Fig 3). We also found that inhibition of mTOR with rapamycin blocked the upregulation of Id2 induced by TNFα (Fig 4). We further found that treatment with TNFα and RANKL activated Akt and mTOR through PI3K (Fig 5). These results suggested that cyst fluid TNFα might act through PI3K-Akt mediated activation of mTOR to increase the levels of Id2 to regulate cyst lining epithelial cell proliferation during cyst development, which might be one of the reasons that rapamycin could inhibit cyst lining epithelial cell proliferation in *Pkd1* knockout mouse models [38]. Since rapamycin treatment also induces apoptosis of cystic epithelial cells [38], whether rapamycin-induced apoptosis is through TNFα and TNF receptor 1 mediated pro-survival and pro-death pathways, as suggested in our recent publication [4] needs to be further investigated.

RANKL has been found to regulate mammary epithelial cell proliferation via Id2 by triggering marked nuclear translocation of Id2 [12], implying a highly specific effect of NF-κB signaling on Id2 function. RANKL was unable to induce expression of Id2 mRNA and protein in mammary epithelial cells, however, RANKL triggered Id2 nuclear transport, which could be completely abrogated in the presence of the Cdk2 inhibitor, roscovitine [12]. We found that TNFα and RANKL treatment increased Id2 protein expression and Id2 nuclear localization in renal epithelial cells (Figs 6 and 7), and roscovitine blocked TNFα and RANKL-induced Id2 nuclear transportation in renal epithelial cells (Fig 7). Roscovitine was previously shown to slow down cyst formation and improve renal function [40]. Our results now suggest that the mechanism by which roscovitine acts may be through Id2 nuclear import to regulate renal epithelial cell proliferation in PKD mouse models.
